# Pervasiveness of intronless genes expressed in haploid germ cell differentiation

**DOI:** 10.1002/rmb2.12385

**Published:** 2021-05-04

**Authors:** Hiromitsu Tanaka, Akira Tsujimura

**Affiliations:** ^1^ Lab. of Molecular Biology Faculty of Pharmaceutical Sciences Nagasaki International University Sasebo Japan; ^2^ Department of Urology Juntendo University Hospital Urayasu Japan

**Keywords:** chromosome, meiosis, retrotransposition, sperm, testis

## Abstract

**Background:**

cDNA libraries derived from the brain and testis contain genes that encode almost all proteins. The brain is composed of various differentiated cells, and the testis also contains various differentiated cells, such as germ cells, and somatic cells that support germ cell differentiation, such as Sertoli and Leydig cells. Many genes appear to be expressed due to tissue complexity.

**Methods:**

The Genome Project has sequenced the entire genomes of humans and mice. Recent research using new gene analysis technologies has found that many genes are expressed specifically in male germ cells.

**Main findings (Results):**

Functional intronless genes are significantly enriched in haploid germ cell‐specific genes.

**Conclusion:**

Functional intronless genes associated with fertility are more likely to be inherited in haploid germ cells than in somatic cells.

## Graphical abstract

More genes expressed in the testis have been cloned, and those expressed specifically in germ cells have been analyzed. The results showed that numerous intronless genes are specifically expressed and have a function in germ cells. In this paper, we consider the reasons why many intronless genes are expressed during spermatogenesis.

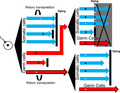

## 1 INTRODUCTION

In the fertilized egg, the DNA inherited from the egg and sperm is replicated, after which the egg undergoes cell proliferation. During the differentiation and proliferation of one generation of germ cells, approximately 70 base substitutions occur in chromosomal DNA.^1^ Among germ cells in which base substitutions occur during differentiation and proliferation due to mitosis, some haploid cells escape base substitutions after meiosis.^2^ Somatic cells die after continual cell division, whereas germ cells continue to divide by accurately replicating their DNA, as homologous recombination and meiosis are essential for cellular maintenance and passing genetic traits to offspring.^2^ We cloned the genes specifically expressed in haploid male germ cells to compare the characteristics of germ cells and somatic cells.^3^ The Genome Project revealed that genes encoding about 25,000 proteins are expressed in humans and the mouse. Recently, 2017 intronless genes were identified in the mouse genome.^4^ It was reported that 99% of human protein‐coding genes align with homologues in the mouse and 80% are clear 1:1 orthologs.^4^ Genes specifically expressed in haploid germ cells have been cloned and their genomic structures analyzed. Genomic structural analyses of 246 cloned genes revealed 51 intronless genes.^5^ These results revealed that many intronless genes are expressed and function in haploid male germ cells (Table 1).^2^, ^4^, ^5^

**TABLE 1:** Rate of intronless genes in the genes encoding mouse proteins

Cells expressing genes	Number of genes	Number of intronless genes	Rate of intronless genes	References
Germ cell specific	246	51	20%	^5^
Somatic cell specific or non‐specific	≒25 000	1966	8%	^4^

## 2 THE TESTIS AND SPERMATOGENESIS

After puberty, oocytes stop cell division in the first meiotic prophase in the ovary. Some eggs are ovulated and resume cell division, whereas sperm are produced from spermatogonia via meiosis throughout life in the testis, which also produces androgens.^6^ Spermatogenesis occurs in a filamentous tube called the seminiferous tubule within the testis, and germ cell differentiation can be divided into three main stages: proliferation and differentiation of spermatogonia, which are the male germ stem cells, meiosis of spermatocyte cells, and morphogenesis of haploid germ cells. The testis contains seminiferous tubules composed of germ cells surrounded by Leydig cells. In addition, lymphatic vessels, capillaries, and a small number of macrophages are present.^7^, ^8^

The seminiferous tubule wall consists of the basement membrane, connective tissue and surrounding fibroblasts, and myoid cells attached to the outside of the wall. Almost all genes are expressed in the brain and testis.^9^ This is thought to be because gene expression is necessary to support the various differentiated cells of the brain and testis, the latter including differentiated cells from spermatogonia to spermatids and the cells supporting spermatogenesis.

## 3 GENES EXPRESSED IN SPERMATOGENESIS

In mice, the differentiation of male germ cells (spermatogenesis), from spermatogonia to spermatozoa (spermatids) via meiosis, begins immediately after birth and takes approximately 35 days to complete.^6^ With the progress of gene analysis technologies such as differential display, subtracted testis‐specific libraries, and microarray analysis, genes specifically expressed in the testis have been identified.^10^ Approximately 2300 testicular germ cell‐specific genes are distributed across various chromosomes.^11^ By producing gene‐disrupted mice, the functions of these genes were analyzed in vivo; the gene‐disrupted mice produced fertilizable sperm, even if one function was lost in many of these specific genes.^11^ Histones are replaced by transition nuclear proteins (TNPs), which are ultimately replaced by protamine in spermiogenesis. Two TNPs, TNP1 and TNP2, are expressed in sperm nucleation. Loss of either the *TNP1* or *TNP2* gene produces fertilizable sperm, but loss of both genes results in a failure to form fertilizable sperm nuclei. TNP1 and TNP2 partially complement each other.^12^ These results indicate that the roles of individual testicular germ cell‐specific genes may be complemented by the functions of other genes. Spermatogenesis is maintained by the redundancy and complexity of germ cell‐specific genes. From this, it can be understood that the roles of individual genes in the body are complemented by the functions of multiple genes that maintain germ cell differentiation.

## 4 INTRONLESS GENES IN MALE GERM CELLS

Intronless genes are produced by retrotransposition. The Genome Project revealed that human and mouse chromosomes each contain approximately 2000 functional retrotransposed genes,^4^ many of which are expressed in haploid spermatids. Olfactory receptor (OR) genes are intronless genes expressed in somatic cells and sperm.^13^, ^14^ It is believed that the original genes were duplicated to establish multiple different OR genes; however, intronless genes code a wide variety of functions in cells, including germ cells.^2^, ^4^ Twenty‐five genes expressed specifically in spermatogonia are located on the X chromosome,^15^ and retrotransposition of genes, mainly from the X chromosome to autosomal chromosomes, and vice versa, occurs frequently.^16^ For example, two intronless genes on autosomal chromosomes, phosphoglycerate kinase 2 and pyruvate dehydrogenase subunit E2, are thought to be derived from the retrotransposition of ancestral genes on the X chromosome via reverse transcription, which might be a mechanism to avoid X‐chromosome inactivation during spermatogenesis.^17^ However, intronless genes such as phosphoglycerate mutase family member 4 (*PGAM4*) and NFKB activating protein‐like (*NKAP‐L*) are crucial for spermatogenesis and are located on the X chromosome,^18^, ^19^ but the relationship between X‐chromosome inactivation escape and the production of intronless genes remains unclear.

**FIGURE 1:**
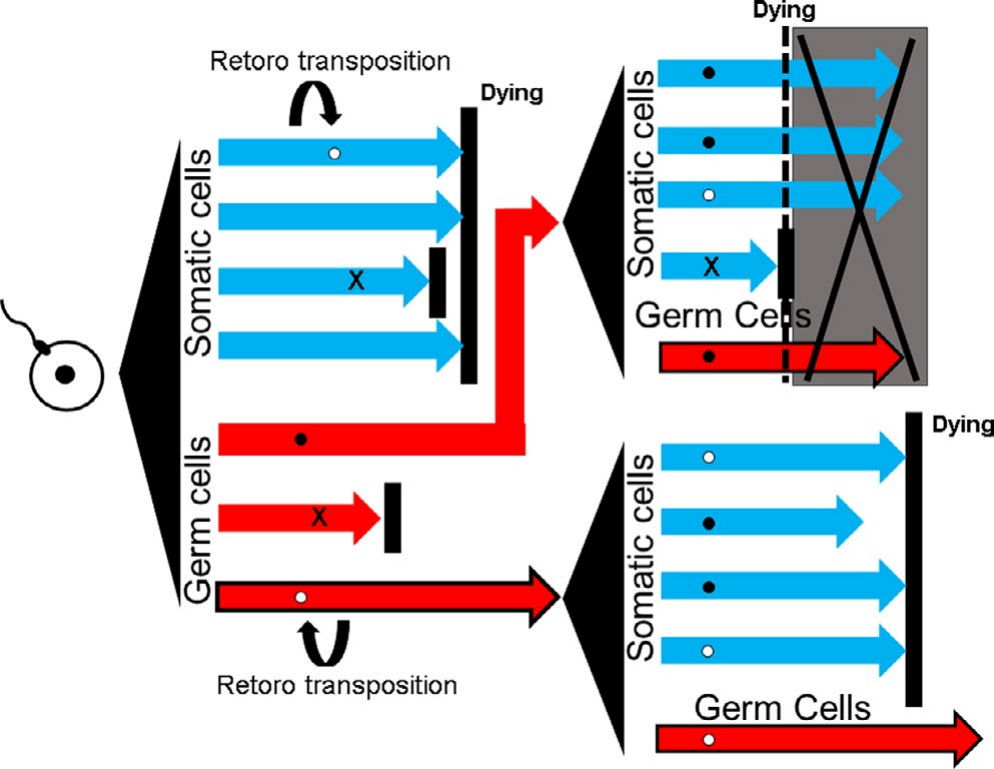
Schematic representation of the heredity of intronless genes in male germ cells. Most multicellular organisms are composed of various differentiated tissues (thick arrow). The cells of the body are divided into somatic (thick blue arrow) and germ (thick red arrow) cells. Retrotransposed genes in somatic cells are not inherited. Alternatively, retrotransposed genes in germ cells are passed on to the next generation unless the gene is incompatible with germ cells. A retrotransposed gene is inherited only when it gains suitable expression and function in the next generation of somatic cells. Circles indicate retrotransposed genes that adapted to cells; black circles indicate inactivated genes; cross marks indicate maladapted transposed genes. A transposed gene in a germ cell will not be inherited by the next generation if any abnormality occurs in cell differentiation in the next generation (upper right). A gene is considered a pseudogene if it is inactive in all cells

Sex chromosomes are thought to play an important role in the production of new genes.^20^ As in autosomal chromosomes, mutations that occur due to gene retrotransposition in female germ cells during ontogenesis can be repaired by homologous recombination between the two X chromosomes, and this process can eliminate harmful mutations. In contrast, homologous recombination does not occur within most regions of the Y chromosome due to large differences between the X and Y chromosomes, except within small homologous regions. Genetic changes that occur in germ cells during ontogenesis might be transmitted to future generations without being repaired by meiosis. Therefore, sex chromosomes with retrotransposed intronless genes may have been passed on to the next generation via male germ cells, eventually spreading to various chromosomes. The presence of intronless germ cell‐specific genes may be explained as follows. Although DNA mutations, including retrotransposons, occur in various cells during ontogenesis, they must occur in specific germ cells during the early stage of embryonic development to be transmitted to future generations. In particular, retrotransposition occurs only in genes transcribed into mRNA. If a gene expressed in germ cells is retrotransposed to another genomic location in addition to being expressed from its original location, it is more likely that the expressed gene will be functional in germ cells than in somatic cells, as the original gene has a defined function in germ cells (Figure 1). Furthermore, spermatids lose most of their cytoplasm, and the histones of most chromosomes are replaced by protamines in the nucleus to reduce splicing.^2^ Therefore, intronless genes may be crucial for gene expression in and regulation of cellular differentiation.

## 5 FUNCTION OF INTRONLESS GENES

**FIGURE 2:**
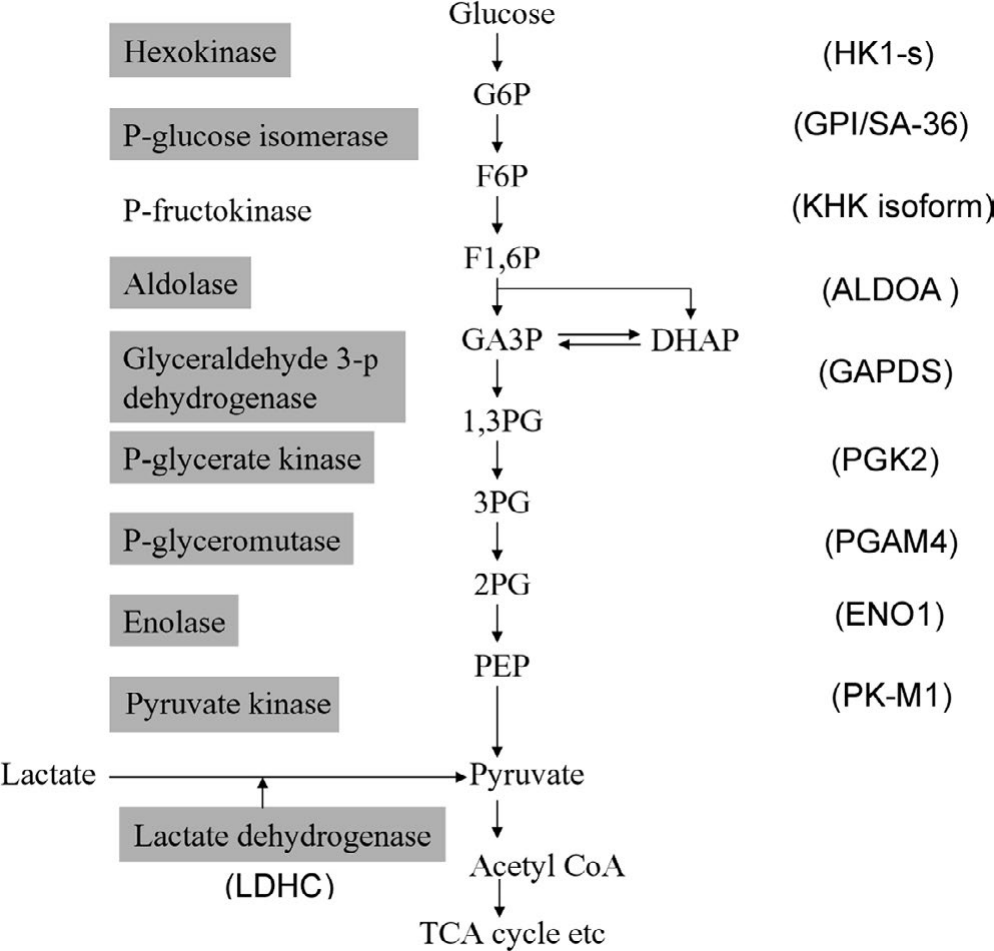
Glycolytic pathway. The name of the enzyme is shown on the left. The isozymes (shadowed) are specifically expressed in haploid cells. The gene name encoding the isozyme is shown in parentheses. Expression of a KHK isoform has been observed in some tissues

Intronless genes include genes related to various cell properties such as cytoskeleton,^21^ signal transduction,^22^ chromatin formation,^23^ and metabolic enzymes^24^ in humans.^25^ In fact, intronless genes encoding spermatid‐specific isozymes, which differ from somatic enzymes, are expressed in spermatids during almost all stages of the glycolytic pathway, from glucose to acetyl‐CoA production (Figure 2).^26^ In addition, *SCOT‐t*, an intronless gene encoding an energy‐metabolizing enzyme, has been identified.^27^ Some genetic polymorphisms associated with male infertility have been identified, and an association between genetic polymorphisms in androgen receptors or the human leukocyte antigen system and infertility has been reported.^28^, ^29^ In a single‐nucleotide polymorphism (SNP) analysis of haploid germ cell‐specific intronless genes in a Japanese cohort,^25^ SNPs in *SCOT‐T* ^30^ and *PGAM 4* ^18^ were found to occur at significantly higher rates in male infertility patients. Four single‐nucleotide polymorphisms were reported in *SCOT‐T*: one in the 3′ noncoding region and three in the coding region causing predicted amino acid substitutions (Table 2).^30^ Homozygotes for the minor allele of the c.854T/G SNP at aa 285 (L285R) were found significantly more often in infertile patients. The minor allele of the c.75G/C SNP at aa 25 (W25C) in *PGAM4* on the X chromosome was also found significantly more often in infertile patients and showed reduced enzymatic activity.^18^ These results indicate that analysis of haploid sperm cell‐specific intronless genes may be useful in understanding infertility.

**TABLE 2:** Prevalence of single‐nucleotide polymorphisms (SNPs) in *SCOT‐T* in infertile or proven fertile populations

SNPs type and position	Genotype	Fertile controls	Infertile cases	Fold increase in infertile cases	Statistical significance
c.113T/C (L38P)^a^	T/T	246 (94)	246 (96)		
	T/C	14 (5.4)	7 (2.7)		
	C/C	1 (0.4)	2 (0.8)	X2	*P* < .54
c.854T/G (L285R)^a^	T/T	208 (80)	204 (80)		
	T/G	50 (19)	39 (15)		
	G/G	3 (1.1)	12 (4.7)	X4	*P* < .018
c.1055C/G (T352M)^a^	C/C	251 (96)	238 (93)		
	C/T	8 (3.1)	11 (4.3)		
	T/T	2 (0.8)	6 (2.4)	X3	*P* < .17
c.1651T/C	T/T	209 (80)	205 (80)		
	T/C	49 (19)	38 (15)		
	C/C	3 (1.2)	12 (4.8)	X4	*P* < .018

^a^ Parentheses indicate amino acid substitution.

*HASPIN* is another characteristic intronless gene of interest.^31^ *HASPIN* is predominantly expressed in male germ cells and regulates oocyte meiotic maturation.^32^ HASPIN is also slightly expressed in somatic cells, where it phosphorylates histone H3 threonine 3 and plays an important role in chromosomal distribution during cell division.^33^ However, no specific phenotype was observed in *HASPIN* gene‐disrupted mice.^34^ Recently, experiments using HASPIN inhibitors have shown that the proliferation of cultured cancer cells was specifically suppressed,^35^ as was intestinal polyp development in *Apc^min/+^* mice in a familial colon tumor disease model in vivo.^36^ These results indicate that the function of HASPIN is compensated by other molecules in normal somatic cells, but it may be an essential molecule for the proliferation of cancer cells. Analysis of intronless genes, including HASPIN, may be useful in researching both infertility and cancer treatments. Interestingly, although *HASPIN* exists only as an intron‐containing gene in fish, it exists as both an intronless and intron‐containing gene in amphibians. In mammals, intronless *HASPIN* exists within an intron of the integrin αE gene,^37^ whereas intron‐containing *HASPIN* exists as a pseudogene. Although intronless *HASPIN* is present in some reptile and bird species, the genes surrounding *HASPIN* on the chromosome vary. Analysis of the chromosomal variations of intronless *HASPIN* may be useful in understanding the evolution of living organisms.

## 6 FUTURE PERSPECTIVES

Analysis of the function of germ cell‐specific intronless genes will shed light on the causes of male infertility. Additionally, the primary structures of the genomes of various organisms will be clarified and compared, together with the origins of intronless genes. These results may further elucidate the evolution of the genome and its essential features.

## CONFLICT OF INTEREST

The authors confirm that there are no conflicts of interest with the contents of this review.
